# m^6^A-modified circRNA MYO1C participates in the tumor immune surveillance of pancreatic ductal adenocarcinoma through m^6^A/PD-L1 manner

**DOI:** 10.1038/s41419-023-05570-0

**Published:** 2023-02-14

**Authors:** Hua Guan, Kun Tian, Wei Luo, Mingfei Li

**Affiliations:** 1 Department of Health Management, Sichuan Provincial People’s Hospital, University of Electronic Science and Technology of China, Chengdu, Sichuan China; 2 Department of Stomatology, Sichuan Provincial People’s Hospital, University of Electronic Science and Technology of China, Chengdu, Sichuan China; 3 Department of Hepatobiliary Surgery, Sichuan Provincial People’s Hospital, University of Electronic Science and Technology of China, Chengdu, Sichuan China

**Keywords:** Immunogenetics, Immunoediting

## Abstract

Emerging evidence indicates the critical roles of N^6^-methyladenosine (m^6^A) modification in human cancers. Herein, our work reported that a novel m^6^A-modified circRNA from the MYO1C gene, circMYO1C, upregulated in the pancreatic ductal adenocarcinoma (PDAC). Our findings demonstrated that circMYO1C is highly expressed in PDAC tissues. Functionally, circMYO1C promoted the proliferation and migration of PDAC cells in vitro and its silencing reduced the tumor growth in vivo. Mechanistically, circMYO1C cyclization was mediated by m^6^A methyltransferase METTL3. Moreover, methylated RNA immunoprecipitation sequencing (MeRIP-seq) unveiled the remarkable m^6^A modification on PD-L1 mRNA. Moreover, circMYO1C targeted the m^6^A site of PD-L1 mRNA to enhance its stability by cooperating with IGF2BP2, thereby accelerating PDAC immune escape. In conclusion, these findings highlight the oncogenic role of METTL3-induced circMYO1C in PDAC tumorigenesis via an m^6^A-dependent manner, inspiring a novel strategy to explore PDAC epigenetic therapy.

## Introduction

Pancreatic ductal adenocarcinoma (PDAC) is one of the most malignant tumors with high mortality and tens of thousands of newly diagnosed cases are reported by global statistics [1, 2]. Worldwide, the widespread application of screening has reduced the global morbidity and mortality associated with PDAC [3]. Although great progress have done in PDAC treatment, the 5-year survivals are still fabulously less than 6% [4, 5]. Until now, the molecular mechanism underlying the progression of PDAC is still unclear, thus it would be highly ponderable for the identification of early diagnosis and novel therapeutic targets establishment.

Circular RNAs (circRNAs) is a newly identified special class of non-coding RNA (ncRNA) that plays critical regulatory roles in the progression of cancer [6, 7]. In the tumorigenesis of PDAC, it has been demonstrated that circRNAs regulate the pathophysiological process through the diverse molecular mechanism. For instance, circRNA circCUL2 specifically expresses in cancer-associated fibroblasts and is significantly correlated with PDAC patients’ poor prognosis. Mechanistically, circCUL2 modulates the miR-203a-3p/MyD88/NF-κB/IL6 axis [8]. Thus, we could conclude that circRNAs significantly regulate the PDAC progression, however, the relationship between the oncogenic role of the circRNA and the pathogenetic mechanism remains unclear.

N^6^-methyladenosine (m^6^A) is the most abundant internal modification of RNAs, including coding and non-coding RNA transcripts. Physiologically, m^6^A plays a great moderating impact on RNA transcripts’ dynamic regulation, referring to splicing, transcribing, and translation. M^6^A modification is deposited by the methyltransferase complex composed of methyltransferase-like-3 (METTL3), METTL14, and WTAP. Inversely, the m^6^A modification is uninstalled by m^6^A demethylases, mainly consisting of FTO and ALKBH5. Existing evidence has indicated that m^6^A modification wildly regulated PDAC progression. For instance, ALKBH5 overexpresses in pancreatic cancer cell line and regulates RNA stabilities of LC25A28 and SLC25A37 through modulating regulators of iron metabolism and underscores the multifaceted role of m6A in pancreatic cancer [9]. Moreover, m^6^A regulator HNRNPC knockdown significantly reduces PDAC cell invasion in vitro and metastasis in vivo, while, HNRNPC overexpression provokes malignant phenotypes of PDAC cells [10]. Although a number of literatures have reported on the profound mechanisms involved in the m^6^A posttranscriptional RNA modification are elusive.

Given that circRNAs are a group of specific back-spliced RNA characterized by circular construction, the m^6^A modification could deservedly occur on the circRNAs. Previous research revealed that thousands of m^6^A-circRNAs were screened by high-throughput sequencing with cell-type-specific expression [11]. Subsequent studies further demonstrated that m^6^A-modified circRNAs regulate cancer progression, including liver cancer [12] and non-small cell lung cancer [13]. However, the role of m^6^A on the biogenesis and function of circular RNAs (circRNAs) biogenesis in PDAC has yet to be addressed. Here, our research found that numerous circRNAs were dysregulated upon METTL3 overexpressed and finally identified a novel circRNA circMYO1C (hsa_circ_0041234, 186 bp) in PDAC. Subsequently, our research performed functional assays to test the role of circMYO1C on PDAC progression and further explore the potential mechanism by which m^6^A modification altered circMYO1C fate.

## Materials and methods

### Tissue samples collection

A total of 60 fresh frozen PDAC tissues and paired normal adjacent tissues were collected from surgery at the University of Electronic Science and Technology of China. The clinical parameters of the included patients were shown in Table 1. None of the patients received any chemoradiotherapy before the operation. The study had been approved by the Ethics Review Board of the University of Electronic Science and Technology of China. All patients were duly informed before the sample collection, and written informed consent was received from each patient.

**Table 1:** The correlations between circMYO1C levels and PDAC patients’ clinicopathological characteristics.

		circMYO1C		*p*
		Low (30)	High (30)	
Age				
<60 year	25	14	11	0.432
≥60 year	35	16	19	
Gender				
Male	34	18	16	0.794
Female	26	12	14	
TNM stage				
I-II	23	17	6	0.003
III-IV	37	13	24	
Differentiation				
Well	11	5	6	0.942
Moderate	37	19	18	
Poor	12	6	6	
Lymph node metastasis				
No	28	18	10	0.038
Yes	32	12	20	

### Cell culture, vector construction, and cell transfection

PDAC cells (PANC-1, Capan-2, BxPC-3, and CFPAC-1) and normal human pancreatic ductal epithelial cell line (HPDE6) were obtained from the American Type Culture Collection (ATCC, Manassas, VA, USA) and Chinese Academy of Sciences Cell Bank (Shanghai, China). Cells were cultured with were cultured in DMEM medium (Gibco, USA) supplemented with 10% of fetal bovine serum (Gibco), 100 U/ml penicillin, and 100 mg/ml streptomycin (Gibco). Cells were cultured in a 5% CO_2_ incubator (Thermo Fisher, USA) in a humidified environment at 37 °C. For the overexpression of circMYO1C, the full-length sequences were amplified and then cloned to the pCD5-ciR vector (Greenseed Biotech, Guangzhou, China). For the silencing of circMYO1C, the lentiviral-based specific short hairpin RNA (shRNA) targeting circMYO1C were designed and synthesized by GenePharm (Shanghai, China). The transfection of plasmids was performed using the Lipofectamine 3000 kit (Invitrogen) PDAC according to the manufacturer’s instructions.

### High-throughput circRNA microarray

To investigate the circRNA expression profile upon METTL3 overexpression, circRNA microarray (ArrayStar, Aksomics) were performed in PDAC according to the instruction. Total RNA was extracted from the PANC-1 cells transfected with METTL3 overexpression plasmids or controls. The RNA was digested with RNase R to remove linear RNAs. Enriched circRNA was amplified and labeled with Arraystar Super RNA Labeling Kit (Arraystar, Rockville, USA). The labeled cRNA was hybridized onto Arraystar Human circRNA Array (8 × 15 K, ArrayStar, Aksomics). CircRNAs with twofold changes and *p* values <0.05 were regarded as significantly different.

### Quantitative real-time polymerase chain reaction (RT-qPCR)

For PCR of circRNA and mRNA, RNA was extracted from PDAC cells and reversely transcribed using Transcriptor First Strand cDNA Synthesis Kit (Roche, Indianapolis, IN). Quantitative PCR was performed using YBR Green PCR Master Mix (Applied Biosystems, Foster City, CA). The relative expression levels were calculated using the 2^−△△CT^ method. The relative level was normalized by GAPDH. Primers were listed in Table S1.

### Actinomycin D assays and RNase R treatment

PDAC cells were seeded in 6-well plates (1 × 10^5^ cells per well) for 24 h. Then, cells were exposed to Actinomycin D (Act D, 2 μg/ml, Sigma) and collected at indicated time points. RNA stability was identified by analyzing the remaining level using qRT-PCR normalized to blank control. For the RNase R treatment, total RNA (2 μg) was incubated with 5 U/μg RNase R (5 U/μg, Epicentre Technologies) and linear RNA or circular RNA transcripts were subsequently analyzed by qRT-PCR.

### Proliferative assays

Proliferative assays were performed to detect the proliferation ability of PDAC cells, including CCK-8 and Ethynyl-2-deoxyuridine (EdU) assays. For CCK-8, PDAC cells were seeded in 96-well plates. Then, 24 h later, cells were administrated with 10 μl of CCK-8 assay kit (Dojindo Japan). The absorbance was measured at 450 nm at the indicated time. For EdU, cells were cultured in 24-well plates and 10 mM EdU was added to each well. After being fixed with 4% formaldehyde and washing, cells were stained with DAPI and visualized using a fluorescent microscope.

### Migration assays

Migration assays were performed to detect the migration of PDAC cells, including wound healing assay and transwell assay. In brief, for the wound healing assay, PDAC cells were seeded in six-well plates at 90% confluence. Cell monolayers were manually wounded by 200 ul pipette tip scraping. After 48 h culture, the migration rate was quantified with measurements of the distance by the following formula: migration distance/original distance. For the transwell migration assay, the transfected PDAC cells were suspended in a serum-free medium and seeded into the upper chambers of the transwell (8-μm pore size). While, full medium with 20% FBS was added to the lower chambers. After incubation, cells on the top chamber were removed and invaded cells on the lower surface were fixed with methanol and stained with 0.1% crystal violet. Photographs were captured by microscope (Olympus).

### MeRIP-qPCR

Total RNA was extracted from PDAC cells using Trizol (Thermo Fisher) and RNA (100 μg) was subjected to 500 μl MeRIP buffer (500 μl). RNA was incubated with anti-N^6^-methyladenosine antibody (ab151230) and rabbit IgG (1 μl) to pull down m^6^A modified circMYO1C. Lastly, the m6A-bound RNA was identified by RT-qPCR.

### RNA immunoprecipitation (RIP)

RIP assay was performed to identify the molecular interaction as previously described [14]. In brief, cells were lysed in complete RIP lysis buffer (Magna RIP Kit, Millipore, MA), and the isolated RNAs were fragmented by sonication and immunoprecipitated with protein A/G magnetic beads conjugated with specific antibodies (anti-METTL3, no. ab195352, Abcam; IGF2BP2, no. 11601–1-AP, Proteintech; anti-Flag, no. 8146, Cell Signaling) or control IgG in RIP Immunoprecipitation buffer (Magna RIP Kit, Millipore, MA) for 2 h at 4 °C. Beads were washed and incubated with Proteinase K to remove proteins. RNAs was extracted and subjected to qRT-PCR using primers and normalizing to input.

### RNA pull-down assay

The pull-down assay was performed as previously described [15]. In brief, the biotinylated circMYO1C probe and oligo probe were designed and synthesized by Genepharm (Shanghai, China). Probes (3 μg) were incubated with Streptavidin magnetic beads (50 μL, Invitrogen) at room temperature for 2 h to generate probe-coated beads. RIP buffer supplemented with a cocktail and agarose beads were used to lyse the cells. Then, the lysate was incubated by a biotinylated probe with streptavidin-coated magnetic beads for 6 h at 4 °C. Finally, the pulled-down protein or RNA was collected and then detected by western blot or qPCR.

### RNA fluorescence in situ hybridization (RNA-FISH)

FISH was performed according to the manufacturer’s instructions. In brief, FAM-labeled circMYO1C probe, cy3-labeled probe IGF2BP2 probe, and DAPI-labeled U6 probes were synthesized by GenePharm (Shanghai, China). In brief, PDAC cells (Capan-2) were fixed and washed in PBS and the suspension was pipetted onto glass slides. After dehydration with ethanol, the hybridization was performed in the dark at 37 °C overnight. After being washed twice, the RNA-FISH was performed using a fluorescent in situ hybridization kit Genepharma (Shanghai, China) according to the manufacturer’s protocol. The images were acquired using a confocal microscope (Olympus).

### Xenograft in vivo mice assay

Male BALB/c nude mice (5–6 weeks) were obtained from Slac Laboratory Animal Center (Shanghai, China) and maintained under pathogen-free conditions. PANC-1 cells (2 × 10^6^ cells suspended in 100 μl PBS) transfected with circMYO1C knockdown (sh-circMYO1C) or controls (sh-NC) were subcutaneously injected into the flank of nude mice. One week later, the tumor size was measured every three days. All procedures were in accordance with the ethical standards and the care of animals and licensing guidelines. The assay had been approved by the Ethics Committee of the University of Electronic Science and Technology of China.

### Statistical analysis

Data were analyzed using GraphPad Prism 8.0 (GraphPad, San Diego, CA, USA) and SPSS 19.0 (Chicago, IL, USA), which were expressed as mean ± SD (standard deviation). The statistical approach comprised a Student’s *t*-test for two independent groups and a one-way analysis of variance (ANOVA) for multiple group comparisons. Correlation analysis was analyzed by Pearson’s correlation coefficient and two-tailed *p* value. *P* < 0.05 was considered significant. All experiments were performed in triplicate.

## Results

### circMYO1C was a METTL3-induced circRNA and upregulated in PDAC

**Fig. 1:**
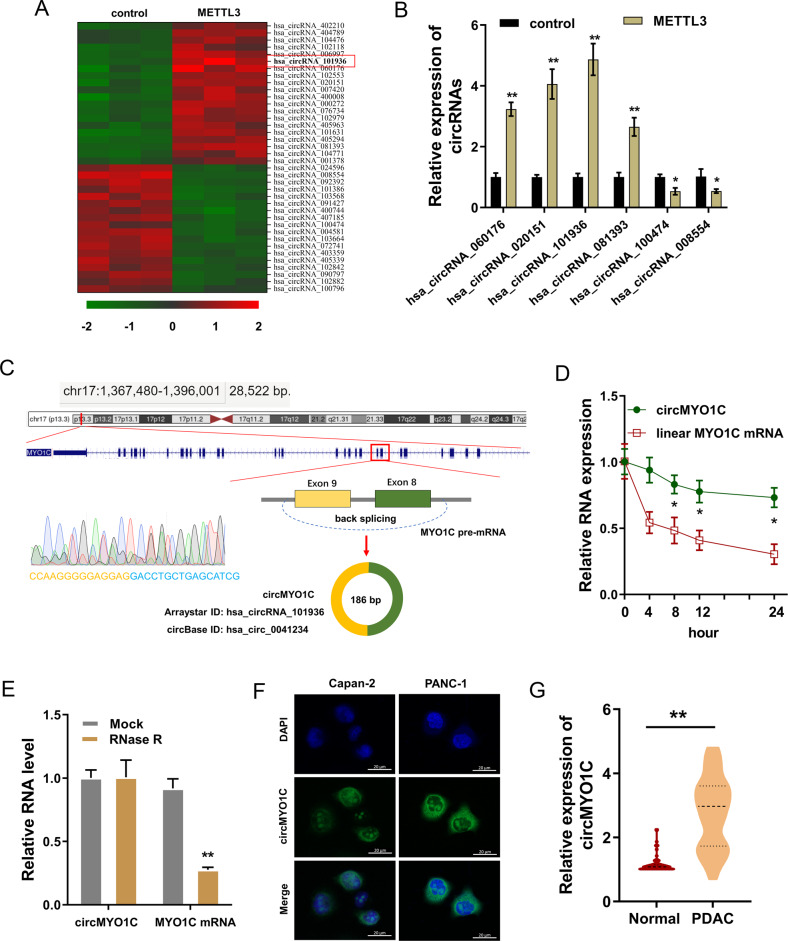
circMYO1C was a METTL3-induced circRNA and upregulated in PDAC. **A** Heatmap of circRNA microarray showed the up or downregulated circRNAs in the METTL3 overexpression transfection versus control transfection in PANC-1 cells. **B** Several candidate circRNAs were quantitatively analyzed by RT-qPCR in PANC-1 cells. **C** The genomic loci of the MYO1C gene and generation of circMYO1C. Sanger sequencing confirmed the head-to-hail splicing site of circMYO1C. **D** Actinomycin D administration assay was performed on PANC-1 cells and then an expression of circMYO1C and linear MYO1C mRNA was detected using RT-qPCR. **E** The RNase R digestion was performed on PANC-1 cells and then an expression of circMYO1C and linear MYO1C mRNA was detected using RT-qPCR. **F** RNA fluorescence in situ hybridization (RNA-FISH) using circMYO1C probes showed the distribution of circMYO1C in PDAC cells. **G** The expression of circMYO1C in the clinical specimens of PDAC patients. *p < 0.05 and **p < 0.01.

To investigate the potential circRNAs regulated by m^6^A modification, we chose METTL3, a crucial m^6^A methyltransferase, to construct the overexpression of METTL3 and its control in PANC-1 cells. CircRNA microarray analysis revealed that numerous circRNAs were dysregulated and we focused on an upregulated circRNA (circMYO1C). CircMYO1C was a 186 bp length transcript derived from the exon9-exon8 of the MYO1C gene. Its ID was hsa_circ_0041234 (circBase) and hsa_circRNA_101936 (ArrayStar, Aksomics) (Fig. 1A). Moreover, several candidate circRNAs were quantificationally validated by RT-PCR, and results demonstrated that circMYO1C showed a higher expression as compared to control group (Fig. 1B). CircMYO1C was an exonic circRNA spliced from MYO1C gene exon9-exon8 via back splicing, thus named as circMYO1C, which was confirmed by Sanger sequencing (Fig. 1C). To detect the stability of circMYO1C, PANC-1 cells were treated with RNase R (RNA synthesis inhibitor) and actinomycin D (DNA repair inhibitor). The half-life time of circMYO1C was longer than MYO1C mRNA (Fig. 1D). qRT-PCR results showed that circMYO1C was more capable of resistance to RNase R digestion (Fig. 1E). RNA fluorescence in situ hybridization (RNA-FISH) displayed that the circMYO1C distributed in the cytoplasm of PDAC cells (Fig. 1F). In the clinical specimens of PDAC patients, the quantitative analysis found that the expression of circMYO1C upregulated in PDAC as compared to normal controls (Fig. 1G). Taken together, these findings indicated that circMYO1C was a METTL3-induced circRNA and upregulated in PDAC.

### circMYO1C promoted the proliferation and migration of PDAC cells

**Fig. 2:**
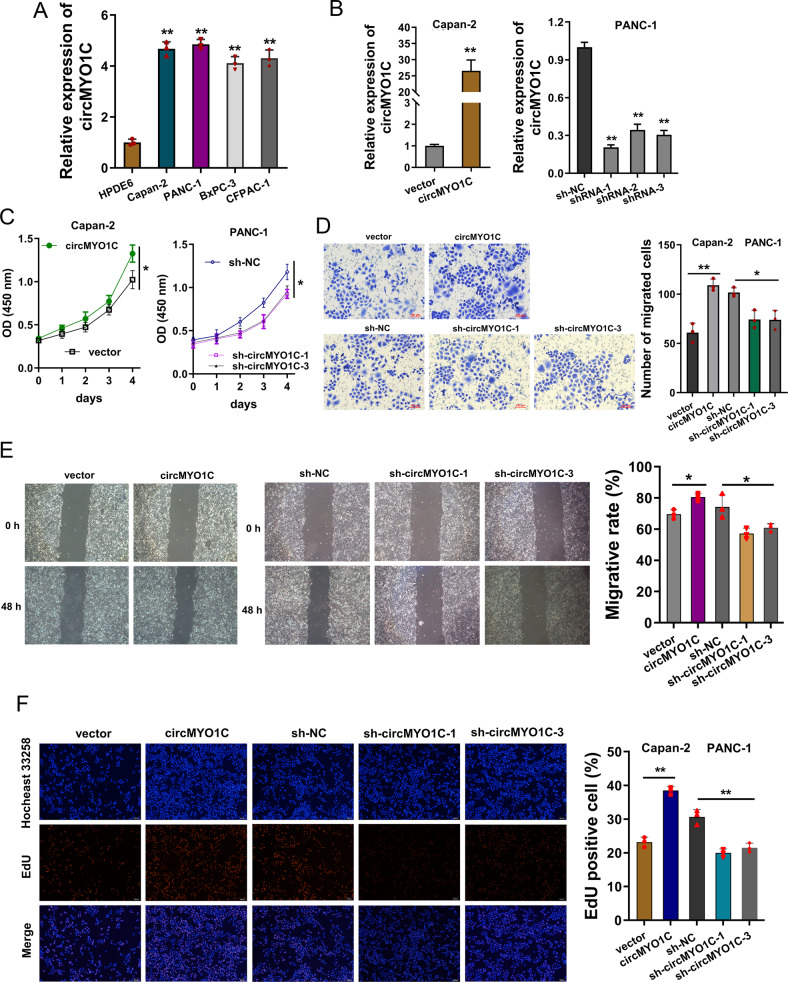
circMYO1C promoted the proliferation and migration of PDAC cells. **A** RT-qPCR indicated the circMYO1C expression in PDAC cells (PANC-1, Capan-2, BxPC-3, CFPAC-1) normalized to normal cells (HPDE6). GAPDH acted as the internal control. **B** pcDNA vector (Capan-2 cell line) and short hairpin RNA (shRNA1/2/3) stable transfection (PANC-1 cell line) were performed following RT-qPCR quantitative analysis. **C** CCK-8 viability assay was performed for PDAC cells’ proliferation using short hairpin RNA (shRNA1/3) or circMYO1C overexpression. **D** Transwell migration assay was performed for the migrative ability of PDAC cells. Migrated cells were counted using short hairpin RNA (shRNA1/3) or circMYO1C overexpression. **E** Wound healing assay was performed for the migrated distance of PDAC cells after 48 h using short hairpin RNA (shRNA1/3) or circMYO1C overexpression. **F** Ethynyl-2-deoxyuridine (EdU) incorporation assay was performed for DNA synthesis using short hairpin RNA (shRNA1/3) or circMYO1C overexpression. EdU-positive cells were counted. *p < 0.05 and **p < 0.01.

In PDAC cells, we found that the circMYO1C expression was upregulated as compared to normal cells (Fig. 2A). To investigate the functions of circMYO1C on PDAC cells, the enforced overexpression and silencing of circMYO1C were respectively transfected into Capan-2 cells (vector, circMYO1C overexpression) and PANC-1 cells (sh-NC, sh-circMYO1C) (Fig. 2B). CK-8 proliferation assay indicated that circMYO1C overexpression promoted the proliferative ability of PDAC cells and circMYO1C silencing repressed the proliferation (Fig. 2C). Migration assay indicated that circMYO1C overexpression promoted the migrative ability of PDAC cells and circMYO1C silencing repressed the migration (Fig. 2D). Wound healing assay unveiled that enhanced ircMYO1C PDAC elerated the migrative ability, while the knockdown of circMYO1C inhibited the migration (Fig. 2E). Ethynyl-2-deoxyuridine (EdU) incorporation assay illustrated that circMYO1C overexpression promoted the DNA synthesis, while the knockdown of circMYO1C repressed the DNA synthesis (Fig. 2F). Overall, these data demonstrated that circMYO1C promoted the proliferation and migration of PDAC cells.

### METTL3 induced the circularization of circMYO1C via m6A-modified flanking intron manner

**Fig. 3:**
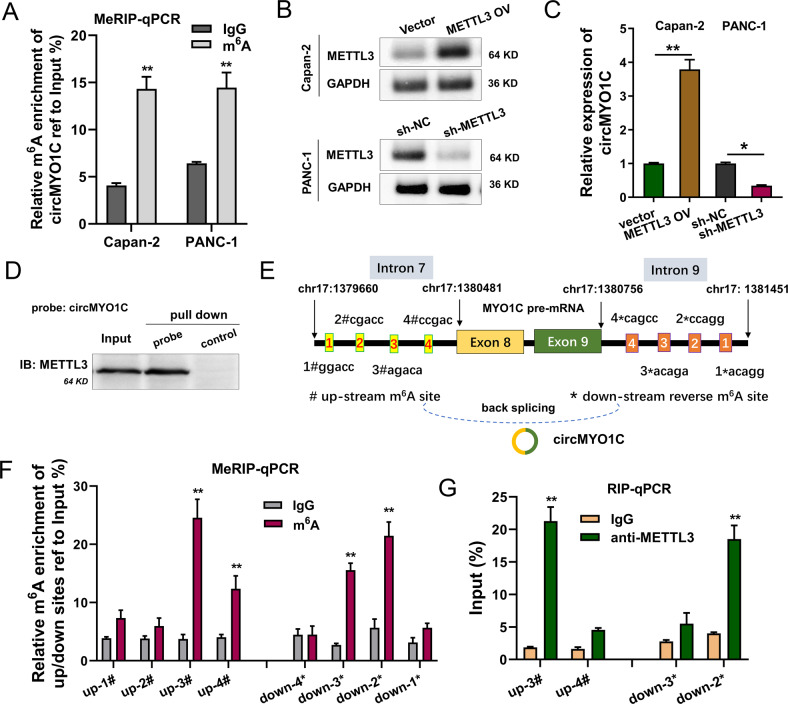
METTL3 induced the circularization of circMYO1C via m^6^A-modified flanking intron manner. **A** MeRIP-PCR analysis of m^6^A enrichment of circMYO1C in the PDAC cells (Capan-2, PANC-1). **B** Western blot analysis detected the METTL3 production in Capan-2 cells transfected with METTL3 overexpression and in PANC-1 cells transfected with METTL3 silencing. **C** RT-qPCR analysis detected the circMYO1C level in Capan-2 cells or PANC-1 cells transfected with METTL3 overexpression or silencing. **D** The interactions between METTL3 and circMYO1C was identified by RNA pull-down assays in Capan-2 cells using biotin-labeled circMYO1C probes. Western blots identified the pulled METTL3 protein. **E** Genomic schematic diagram displayed the potential m^6^A-modified sites in the upstream (intron 7) and reverse m^6^A sequence in downstream (intron 9). **F** MeRIP-PCR analysis detected the m6A enrichment of ruptured sequences n upstream (up-1#, up-2#, up-3#, up-4#) and downstream (down-1*, down-2*, down-3*, down-4*). **G** RIP-qPCR assay using anti-METTL3 or anti-IgG illustrated the molecular binding of METTL3 with an upstream site (up#) and downstream site (down*). *p < 0.05 and **p < 0.01.

The latest research shows that METTL3 could regulate the biogenesis of circRNAs, thus we investigate the interaction between METTL3 and circRNAs. Given that circMYO1C was upregulated in the sequencing upon METTL3 overexpression, we proposed a hypothesis that METTL3 induced the circularization of circMYO1C. Firstly, MeRIP-qPCR analysis found that the m^6^A-modified enrichment was higher in the PDAC cells (Capan-2, PANC-1) (Fig. 3A). Then, the upregulated or downregulated METTL3 construction was performed, whose transfection efficiency was identified by western blot (Fig. 3B). RT-qPCR analysis fund that circMYO1C level was upregulated upon METTL3 overexpression transfection, while circMYO1C level was repressed upon METTL3 silencing (Fig. 3C). RNA pull-down following western blot assays further verified that METTL3 could interact with circMYO1C in Capan-2 cells (Fig. 3D). In terms of the genome location, there were several potential m^6^A-modified sites in the upstream (intron 7) and reverse m^6^A sequence in downstream (intron 9), suggesting the binding sites of METTL3 on circMYO1C flanking sequences (Fig. 3E). MeRIP-PCR analysis of m^6^A enrichment of ruptured sequences showed that there were remarkable m^6^A modification in upstream (up-3#, up-4#) and downstream (down-2*, down-3*) (Fig. 3F). To investigate the binding of METTL3 targeting m^6^A modified flanking sites, RIP-qPCR assay using anti-METTL3 or anti-IgG was performed. Results illustrated that there was one upstream site (up-3#) and one downstream site (down-2*) that displayed the molecular binding with METTL3 (Fig. 3G). Overall, these findings unveiled METTL3 induced the circularization of circMYO1C via m^6^A-modified flanking intron manner.

### circMYO1C promoted the PD-L1 mRNA stability

**Fig. 4:**
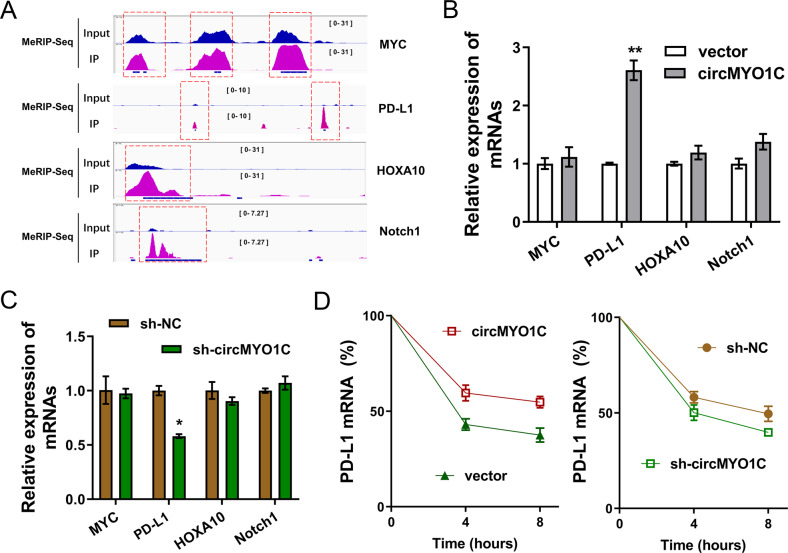
circMYO1C promoted the PD-L1 mRNA stability. **A** IGV (Integrative Genomics Viewer, https://igv.org/) tools for the MeRIP-Seq data showed the m^6^A modification in the genomic location of several candidate genes (MYC, PD-L1, HOXA10, Notch1). RT-qPCR analysis revealed the PD-L1 mRNA level in **B** Capan-2 cells transfected with circMYO1C overexpression and in **C** PANC-1 cells transfected with sh-circMYO1. **D** RNA stability assay using Act D administration showed the PD-L1 mRNA level in Capan-2 cells transfected with circMYO1C overexpression and PANC-1 cells transfected with sh-circMYO1. *p < 0.05 and **p < 0.01.

MeRIP-Seq revealed that several candidate genes (MYC, PD-L1, HOXA10, Notch1) demonstrated the m^6^A modification in their genomic location (Fig. 4A). Then, RT-qPCR analysis revealed that circMYO1C overexpression significantly upregulated the PD-L1 mRNA level, while circMYO1C knockdown reduced the expression of PD-L1 mRNA (Figs. 4B, C). Meanwhile, the other candidates showed no significant change. Moreover, RNA stability assay using Act D administration showed that circMYO1C overexpression remarkably increased the PD-L1 mRNA stability and circMYO1C knockdown repressed the PD-L1 mRNA stability (Fig. 4D). Taken together, these findings found that PD-L1 exhibited the m^6^A modification and circMYO1C promoted the PD-L1 mRNA stability.

### circMYO1C interacted with PD-L1 through m^6^A reader IGF2BP2

**Fig. 5:**
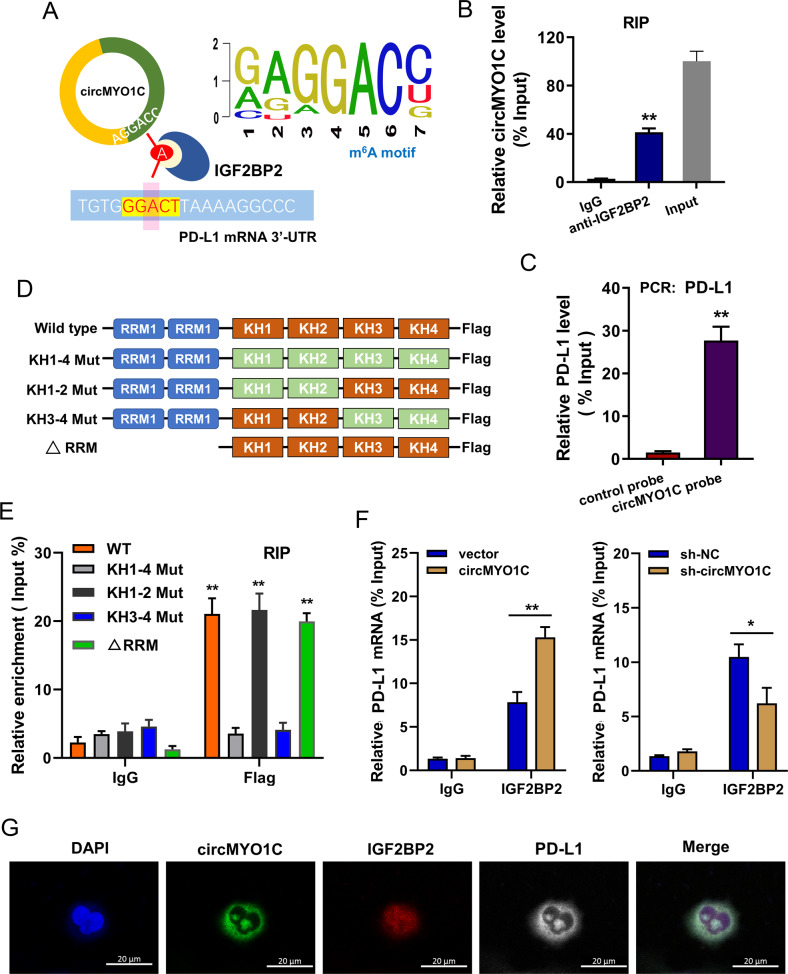
circMYO1C interacted with PD-L1 through m^6^A reader IGF2BP2. **A** Schematic diagram indicated the potential m^6^A-modified sites with both circMYO1C junction sites and PD-L1 3’-UTR. IGF2BP2 motif was GAGGAC (up). **B** RNA binding protein immunoprecipitation (RIP) analysis detected the circMYO1C expression enriched by anti-IGF2BP2 antibody or control IgG precipitation. **C** RNA pull-down assay following PCR detected the PD-L1 mRNA level pulled by the circMYO1C probe. **D** FLAG-tagged full-length and truncated IGF2BP2 mutants were constructed, including 2 RNA-recognition-motif (RRM) domains and 4 K homology (KH) domains. Mutation of GxxG to GEEG was constructed in the KH domains. **E** RIP-qPCR analysis revealed the relative enrichment of circMYO1C with truncated IGF2BP2 protein complex compared to the input control. (**F**) RIP-qPCR analysis revealed the interaction of IGF2BP2 with PD-L1 mRNA upon circMYO1C overexpression (circMYO1C) or knockdown (sh-circMYO1C). **G** RNA-FISH showed the colocalization of circMYO1C, IGF2BP2, and PD-L1 in Capan-2 cells. DAPI presents the nucleus. *p < 0.05 and **p < 0.01.

Previous research has reported that circRNA could regulate its target mRNA stability, which is mediated by m^6^A reader IGF2BP2 [16]. For the further mechanism by which circMYO1C regulates the stability of PD-L1 mRNA, our research put forward a hypothesis that circMYO1C might interact with PD-L1 through m^6^A reader IGF2BP2. Analytical investigation revealed that IGF2BP2 shared the potential m^6^A modified sites with both circMYO1C junction sites and PD-L1 3′-UTR (Fig. 5A). RNA binding protein immunoprecipitation (RIP) analysis proved that, compared with the control IgG, circMYO1C expression was enriched in the anti-IGF2BP2 antibody precipitation (Fig. 5B). RNA pull-down assay using circMYO1C probe confirmed that circMYO1C specifically combined with PD-L1 (Fig. 5C). As we know, IGF2BP2 possesses 2 RNA-recognition-motif (RRM) domains and 4 K homology (KH) domains [17, 18]. Thus, the next work for our team was to explore which domain might combine with circMYO1C. FLAG-tagged full-length and truncated IGF2BP3 mutants were constructed (Fig. 5D). RIP analysis proved KH3-KH4 domains of IGF2BP2 specifically interacted with circMYO1C, which was required for its interaction with circMYO1C and PD-L1 mRNA (Fig. 5E). RIP analysis demonstrated that circMYO1C overexpression enhanced the IGF2BP2-PD-L1 interaction, while circMYO1C knockdown reduced the protein-RNA interaction (Fig. 5F). By performing RNA fluorescence in situ hybridization (RNA-FISH) assays, we confirmed the colocalization of endogenously expressed circMYO1C and PD-L1 in the cytoplasm (Fig. 5G). Overall, these findings unveiled that circMYO1C interacted with PD-L1 through m^6^A reader IGF2BP2.

### circMYO1C knockdown repressed the tumor growth in vivo

**Fig. 6:**
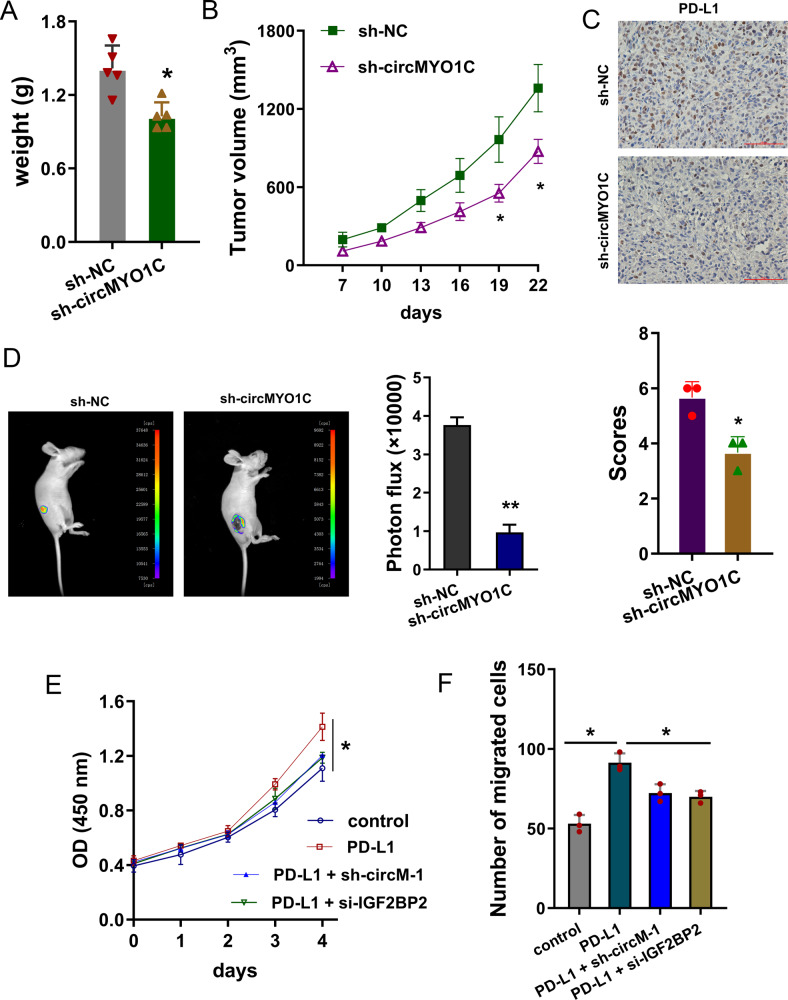
circMYO1C knockdown repressed the tumor growth in vivo. **A** Mice were injected with short hairpin RNA (shRNA-circMYO1C) stable transfection or control groups. Tumor weight was recorded after the mice sacrifice. **B** Tumor volume was recorded every three days and calculated using the formula (0.52 × length × width × width). **C** Immunohistochemical staining (IHC) for PD-L1 was performed using the in vivo tissue. **D** Moreover, the PDAC cells were transfected with firefly luciferase-labeled vectors. The anesthetic mice were monitored using bioluminescence in vivo imaging system. **E** CCK-8 viability assay was performed using PANC-1 cells transfected with PD-L1 overexpression (PD-L1) and circMYO1C knockdown (sh-circMYO1C-1) or IGF2BP2 silencing (si-IGF2BP2). **F** Transwell migration assay was performed using PANC-1 cells transfected with PD-L1 overexpression (PD-L1) and circMYO1C knockdown (sh-circMYO1C-1) or IGF2BP2 silencing (si-IGF2BP2). *p < 0.05 and **p < 0.01.

To investigate the role of circMYO1C in vivo, the xenograft in vivo mice assay was performed. Results indicated that circMYO1C knockdown reduced the tumor weight (Fig. 6A) and volume (Fig. 6B). Immunohistochemical staining (IHC) showed that the PD-L1 protein was decreased upon circMYO1C knockdown (Fig. 6C). Bioluminescence in vivo imaging showed that circMYO1C knockdown repressed the tumor metastasis (Fig. 6D). Rescue experiments were performed and results found that PD-L1 overexpression increased the viability and migration of PDAC cells (Fig. 6E), and co-transfection of circMYO1C knockdown or IGF2BP2 silencing rescued the function (Fig. 6F). Overall, these finding illustrated that circMYO1C knockdown repressed the tumor growth in vivo.

## Discussion

**Fig. 7:**
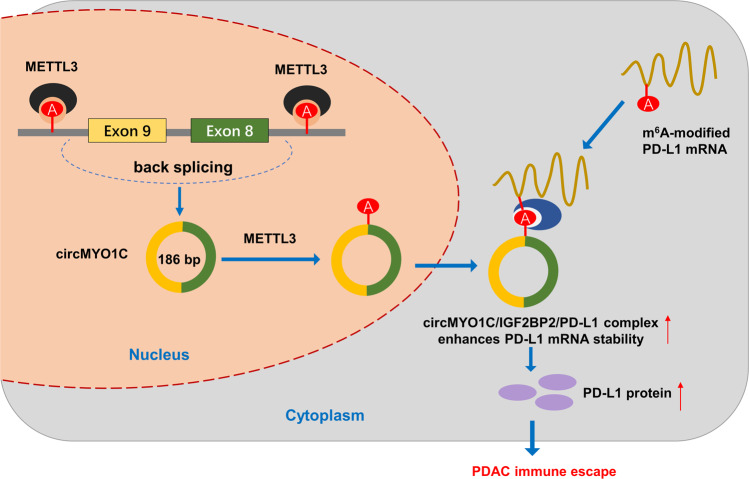
m^6^ A-modified circMYO1C enhances PD-L1 mRNA stability through interacting with IGF2BP2. CircMYO1C highly expressed in PDAC, and circMYO1C cyclization was mediated by m6A methyltransferase METTL3. circMYO1C targeted the m6A site of PD-L1 mRNA to enhance its stability through cooperating with IGF2BP2, thereby accelerating PDAC immune escape.

Emerging evidence has indicated the critical role of epigenetic modification on PDAC tumorigenesis [19, 20]. As an important subgroup of epigenetics, circRNAs play essential roles covering a series of areas, including energy metabolism, proliferation, and metastasis. Moreover, N^6^-methyladenosine (m^6^A) modification regulates the PDAC phenotypic characteristic by controlling RNA fate. On the other hand, circRNA itself is also regulated by m^6^A. Here, our study preliminarily investigates the functions and mechanism of circMYO1C and m^6^A, and further unveils their association with PDAC (Fig. 7).

Although a huge number of circRNAs have been reported and identified to availably modulate the PDAC tumor progression, these circRNAs were only significantly overexpressed/downexpressed in PDAC and did not show association with specific tumor features [21]. The circRNAs with remarkable overexpression or down-expression are usually screened by high-throughput sequencing, and the inclusion criteria for this study is based on their differential expression. Although we all know the fact that higher/lower circRNAs could regulate PDAC, the molecular functions of circRNAs appear to be rather diverse and are, in most cases, only poorly understood.

Our research doesn’t just look at this traditional area that circRNA modify PDAC via miRNAs sponge, and we also turned the focus of research into its biogenesis. We found that m^6^A methyltransferase METTL3 could interact with circMYO1C via m^6^A-dependent manner. Importantly, METTL3 increased the enrichment of circMYO1C, which give us an important insight that the investigation of circRNA biogenesis could provide additional knowledge regarding PDAC tumorigenesis rather than downstream cascade reaction.

To date, emerging novel findings about m^6^A and PDAC have been authenticated by researchers. Given the identified roles of METTL3 in PDAC, we related the METTL3 functions with circRNA biogenesis to explore the possibility of m^6^A-circRNA interaction. To test the interaction between METTL3 and circRNAs, we performed the circRNA screening with the treatment of METTL3 overexpression. Results found that numerous circRNAs were screened out, and we focused on a novel circRNA circMYO1C. Functional experiments revealed that circMYO1C promoted the proliferation and migration of PDAC cells, indicating the oncogenic roles of circMYO1C.

During the progression of the splicing reaction, pre-mRNAs could produce covalently closed circRNAs. The highlight that attracted us most was the cyclization-promoting of METTL3 for circMYO1C. Given that circMYO1C was a METTL3-related circRNA with an m^6^A modification site, we aimed to investigate the potential role of METTL3 on circMYO1C back splicing. Results indicated that METTL3 overexpression indeed upregulated the level of circMYO1C, suggesting the cyclization-promoting role of METTL3 for circMYO1C. Moreover, MeRIP-Seq revealed that there was a remarkable m^6^A modified site on the 3′-UTR of PD-L1 and circMYO1C enhanced the stability of PD-L1 mRNA. Further research illustrated that circMYO1C interacted with PD-L1 through m^6^A reader IGF2BP2, and circMYO1C specifically interacted with the KH3-KH4 domains of IGF2BP to interact with PD-L1 mRNA.

The latest research about m^6^A and circRNA have partially revealed the interesting field [22]. Dan Xie [16] (2019) reported that an m^6^A-modified circRNA circNSUN2 frequently upregulated in tumor tissues from colorectal carcinoma patients with liver metastasis. The m^6^A modification of circNSUN2 promotes its export to the cytoplasm mediated by YTHDC1. Moreover, m^6^A-modified circNSUN2 enhances the stability of HMGA2 mRNA through m^6^A interaction bindings, forming circNSUN2/IGF2BP2/HMGA2 RNA-protein ternary complex. Besides, a novel METTL3-induced circRNA, circRNA circ1662, exhibits significantly higher expression in colorectal carcinoma (CRC) and circ1662 could promote cellular invasion and migration in vitro and in vivo. Mechanistically, METTL3 binds the flanking sequences of circ1662 and installing the m^6^A modification, thereby inducing circ1662 biogenesis [23]. Thus, the evidence indicates that m^6^A modification of circRNA could effectively regulate the pathophysiological process, suggesting the novel m^6^A-circRNA manner. Here, our finding reported a novel m^6^A-modified circRNA circMYO1C in PDAC, revealing a novel insight about m^6^A and circRNA and immune surveillance on PDAC.

## Conclusions

In conclusion, our findings indicated that circMYO1C significantly upregulated PDAC tissues and acted as an oncogenic factor for PDAC. These results suggest that circMYO1C/IGF2BP2/PD-L1 form an RNA-protein complex to PDAC, elevates the proliferation and migration of PDAC cells (Fig. 7). Our findings provide additional evidence for the m^6^A-circRNA manner in PDAC tumorigenesis, providing a novel insight for the circRNA biological effects.

## Supplementary information

Original Data File

aj-checklist

Table S1.
