# Supplementary material for: m6A-modified circRNA MYO1C participates in the tumor immune surveillance of pancreatic ductal adenocarcinoma through m6A/PD-L1 manner
Source: Cell Death Dis. 2023 Feb 14;14(2):120. doi: 10.1038/s41419-023-05570-0 (PMC9925427; doi:10.1038/s41419-023-05570-0)
Supplement: Supplementary file 3 — Table S1. [file 41419_2023_5570_MOESM3_ESM.docx]

**supplement Table S1**. Primers sequences for qRT-PCR and sequences of shRNA.

|  | Sequences |
| --- | --- |
| circMYO1C | forward, 5’-CTCAAGTATCTGACCAGGCTCC-3’  reverse, 5’-TTGCCCAAATGAAGGACGCT-3’ |
| IGF2BP2 | forward, 5’- AGCTAAGCGGGCATCAGTTTG-3’  reverse, 5’- CCGCAGCGGGAAATCAATCT-3’ |
| PD-L1 | forward, 5’- TGGCATTTGCTGAACGCATTT-3’  reverse, 5’-TGCAGCCAGGTCTAATTGTTTT-3’ |
| sh-circMYO1C-1 | 5’-GGAGGAGGACCTGCTGAGCAT-3’ |
| sh-circMYO1C-2 | 5’- GGGAGGAGGACCTGCTGAGCA-3’ |
| sh-circMYO1C-3 | 5’-CGCCAAGGGGGAGGAGGACCT-3’ |
| circMYO1C probe | 5’-AGCGAATAGACACCTCTCCCACCTGACATC  TGATGAAGACTGTGTCTGCGG-3’ |
| GAPDH | forward, 5’- GGAGCGAGATCCCTCCAAAAT-3’  reverse, 5’- GGCTGTTGTCATACTTCTCATGG-3’ |
